# Plasma p-tau181 and p-tau217 in discriminating PART, AD and other key neuropathologies in older adults

**DOI:** 10.1007/s00401-023-02570-4

**Published:** 2023-04-09

**Authors:** Lei Yu, Patricia A. Boyle, Shorena Janelidze, Vladislav A. Petyuk, Tianhao Wang, David A. Bennett, Oskar Hansson, Julie A. Schneider

**Affiliations:** 1 Rush Alzheimer’s Disease Center, Rush University Medical Center, 1750 W Harrison Street, Suite 1000, Chicago, IL 60612 USA; 2 Department of Neurological Sciences, Rush University Medical Center, Chicago, IL USA; 3 Department of Psychiatry and Behavioral Sciences, Rush University Medical Center, Chicago, IL USA; 4 Clinical Memory Research Unit, Department of Clinical Sciences, Lund University, Malmö, Sweden; 5 Pacific Northwest National Laboratory, Richland, WA USA; 6 Memory Clinic, Skåne University Hospital, SE-205 02 Malmö, Sweden; 7 Department of Pathology, Rush University Medical Center, Chicago, IL USA

**Keywords:** Plasma p-tau217, p-tau181, Alzheimer’s disease, PART, Mixed pathologies

## Abstract

We examined whether plasma p-tau181 and p-tau217 are specific biomarkers of pathologically confirmed Alzheimer’s disease (AD). In particular, we investigated the utility of plasma p-tau for differentiating AD from primary age-related tauopathy (PART), as well as AD with mixed pathologies. Data came from 269 older adults who participated in the Religious Orders Study or the Rush Memory and Aging Project. Blood samples were collected during annual clinical evaluations. Participants died and underwent brain autopsy. P-tau181 and p-tau217 were quantified in the plasma samples proximate to death (average interval before death: 1.4 years) using Lilly-developed MSD immunoassays. Uniform neuropathologic evaluations assessed AD, PART, and other common degenerative and cerebrovascular conditions. Plasma p-tau217 was more strongly correlated with brain β-amyloid and paired helical filament tau (PHFtau) tangles than p-tau181. Both p-tau markers were associated with greater odds of AD, but p-tau217 had higher accuracy (area under the ROC curve (AUC): 0.83) than p-tau181 (AUC: 0.76). Plasma p-tau markers were almost exclusively associated with AD pathologic indices with the exception of cerebral amyloid angiopathy. Compared to p-tau181, p-tau217 showed a higher AUC (0.82 versus 0.74) in differentiating AD from PART. For either p-tau, we did not observe a level difference between individuals with AD alone and those with mixed AD pathologies. In summary, plasma p-tau181and p-tau217 were specifically associated with AD pathological changes. Further, our data provide initial evidence that p-tau217 may be able to differentiate between AD and PART in individuals with comparable burdens of tau tangle pathology. These results demonstrate the specificity of p-tau217 for AD, supporting its use to identify patients suitable for anti-AD therapies including β-amyloid immunotherapies.

**Supplementary Information:**

The online version contains supplementary material available at 10.1007/s00401-023-02570-4.

## Introduction

Early detection of Alzheimer's disease (AD) pathologies, specifically β-amyloid and hyperphosphorylated tau, facilitates identification of individuals at risk of Alzheimer's disease and related dementias (ADRD). Plasma p-tau181 (phosphorylated at threonine 181) and p-tau217 (phosphorylated at threonine 217) have recently emerged as two promising blood-based biomarkers for AD [12]. Plasma p-tau181 and p-tau217 levels are elevated in Alzheimer’s dementia, as well as among individuals who are cognitively unimpaired but with positive amyloid [12, 27, 44]. Both measures are closely correlated with PET (positron emission tomography) imaging markers of amyloid and tau [16, 34, 43], even though plasma p-tau becomes abnormal before tau-PET [15]. Further, studies have consistently reported high accuracy of plasma p-tau181 and p-tau217 in differentiating individuals with AD from those without AD [16, 22, 29, 34]. Thus, compared to biomarkers that are invasive or expensive, e.g., cerebrospinal fluid (CSF) and neuroimaging, these easily accessible and relatively inexpensive blood-based measures have great potential for transforming the landscape for AD diagnosis [12, 13].

One active area of research on plasma p-tau is to assess their performance in differentiating AD from other neurodegenerative processes. It has been reported that plasma p-tau181 and p-tau217 can accurately differentiate AD from other neurodegenerative disorders including frontotemporal lobar degeneration [21], Parkinson disease and progressive supranuclear palsy [16, 34, 43]. Notably, with a few exceptions [30, 34, 39], many of these studies classified the degenerative disorders based on clinical criteria. Hence the relationships between plasma p-tau and the defining pathologic features of these neurodegenerative conditions are unclear. In particular, it is still unknown whether plasma p-tau can differentiate AD from primary age-related tauopathy (PART), a very common tauopathy in the aging brain. Separately, literatures on whether plasma p-tau can accurately detect AD with mixed pathologies, the leading cause of dementia, just started to emerge [22, 41].

In this work, we extend prior literature by interrogating plasma p-tau181 and p-tau217 in relation to common neuropathologic indices of AD, non-AD degenerative (i.e., Lewy bodies, TDP-43 and hippocampal sclerosis), and cerebrovascular conditions (i.e., infarcts, cerebral amyloid angiopathy (CAA), atherosclerosis and arteriolosclerosis). In addition, we investigated PART, a tauopathy that overlaps with AD in neurofibrillary tangles (NFT) formation but is absent of β-amyloid [10]. Given the close connections between plasma p-tau181 and p-tau217 with β-amyloid [15, 24, 36, 42], we hypothesized that these two p-tau measures differentiate AD from PART. We also investigated the extent to which plasma p-tau discriminated AD when in combination with other pathologies (i.e., mixed AD pathologies).

## Materials and methods

### ﻿The religious orders study and rush memory and aging project

Our study participants came from one of the two ongoing clinicopathologic cohort studies of aging, the Religious Orders Study or the Rush Memory and Aging Project (ROSMAP) [4]. The Religious Orders Study started in 1994 and the participants were older Catholic nuns, priests and brothers from about 40 religious orders across the United States. The Rush Memory and Aging Project started in 1997 and recruits older adults who live in the communities throughout the greater Chicago metropolitan area. All ROSMAP participants were free of known dementia at enrollment, agreed to annual clinical evaluations including blood collection, and brain donation after death. At enrollment, each participant signed an informed consent, an Anatomical Gift Act, as well as an agreement to redistribute data and biospecimen for research purposes. Both studies were approved by an institutional review board of the Rush University Medical Center.

At time of this analysis on December 26, 2022, 3751 ROSMAP participants had completed the baseline evaluation, 2273 had died and 1953 (86%) had undergone brain autopsies. Individuals included in the current work (*N* = 269) were part of a pilot study that examines blood-based AD biomarkers in a convenience sample that were selected based on availability of proximate to death plasma, and to cover a range of cognitive and neuropathologic conditions (Table 1).

**Table 1:** Characteristic of the study participants (*N* = 269)

Age at death, years^†^	91.4 (5.6)
Female^Δ^	189 (70.3%)
Education, years^†^	15.7 (3.3)
*APOE* ε4 carriers^Δ^	55 (21.9%)
Clinical diagnosis^Δ^	
No cognitive impairment	92 (34.2%)
Mild cognitive impairment	87 (32.3%)
Dementia	90 (33.5%)
Alzheimer’s disease^Δ^	194 (74.1%)
β-amyloid load^§^	3.99 (1.03–6.35)
PHFtau tangle density^§^	4.96 (2.25–9.56)
Lewy bodies^Δ^	57 (21.5%)
Hippocampal sclerosis^Δ^	10 (3.8%)
LATE-NC^Δ^	
Stage 0	141 (53.4%)
Stage 1	53 (20.1%)
Stage 2	19 (7.2%)
Stage 3	51 (19.3%)
PART^Δ^	52 (19.3%)
Chronic macroscopic infarcts^Δ^	93 (35.5%)
Chronic microinfarcts^Δ^	89 (34.0%)
Cerebral amyloid angiopathy^Δ^	
None	48 (18.1%)
Mild	144 (54.1%)
Moderate	48 (18.1%)
Severe	26 (9.8%)
Atherosclerosis^Δ^	
None	64 (24.1%)
Mild	135 (50.8%)
Moderate	57 (21.4%)
Severe	10 (3.8%)
Arteriolosclerosis^Δ^	
None	107 (40.5%)
Mild	90 (34.1%)
Moderate	51 (19.3%)
Severe	16 (6.1%)

MMSE mini-mental state examination, LATE-NC Limbic predominant age-related TDP-43 encephalopathy neuropathologic changes, PART primary age-related tauopathy

^†^Mean (standard deviation)

^Δ^*N* (%)

^§^Median (interquartile range)

### Diagnosis of Alzheimer’s dementia

Annual clinical evaluations were conducted at participants’ home by trained nurses and research technicians. The evaluation includes an interview on medical history, documentation of medication use, blood collection, cognitive testing, and neurological examination. The decision rules guiding the diagnosis of Alzheimer’s dementia in the ROSMAP studies were detailed in a prior publication [5]. Briefly, cognition was assessed using a battery of 21 cognitive tests. The cognitive impairment ratings in five cognitive domains were scored by a computer and reviewed by a neuropsychologist, who then provided a clinical judgment on presence of cognitive impairment. Alzheimer’s dementia diagnosis was provided by a clinician after reviewing all available information. The diagnosis follows the 1984 McKhann criteria which require a history of cognitive decline and impairment in memory and at least one other domain [25]. Of note, the ROSMAP studies started in the 1990s and are ongoing. To maintain the diagnostic consistency over time, we have not switched to the criteria recently updated by the National Institute on Aging and the Alzheimer’s Association (NIA-AA) workgroup [26]. A participant with cognitive impairment who did not meet the criteria for dementia was classified as having mild cognitive impairment (MCI). After a participant died, a neurologist reviewed all available clinical data and provided a summary diagnostic opinion on the most likely diagnosis at the time of death [40].

### Brain autopsy and neuropathologic assessments

Brain autopsy occurred on average 9.7 h (SD: 8.0) after a participant died. Brains were removed, weighed and cut coronally into 1-cm slabs. After gross examination for macroscopic infarcts and atherosclerosis, one hemisphere was stored in − 80 °C freezers for biochemical studies, and the other hemisphere was fixed in 4% paraformaldehyde for at least 3 days. Blocks of fixed tissue were dissected from key brain regions for assessing AD, Lewy bodies, limbic-predominant age-related TDP-43 encephalopathy neuropathologic change (LATE-NC), hippocampal sclerosis, PART, as well as vascular conditions of microinfarcts, CAA and arteriolosclerosis. Neuropathologic assessments are blinded to the clinical records.

Tissue blocks from hippocampus, angular gyrus, entorhinal, midfrontal, inferior temporal, calcarine, anterior cingulate and superior frontal cortex were cut into 20 µm sections for immunohistochemistry of β-amyloid and PHFtau tangles [6]. Cortical β-amyloid were labeled with one of three monoclonal antibodies to Aβ, 4G8 (1:9000; Covance Labs, Madison, WI), 6F/3D (1:50; Dako North America Inc., Carpinteria, CA), and 10D5 (1:600; Elan Pharmaceuticals, San Francisco, CA). With the region of interest outlined using a microscope, a sampling grid was placed on the outlined area. Between 20 and 90 images, depending on the size of brain region, were then taken and processed using Image J software. Region-specific β-amyloid load was calculated as the percentage area positive for β-amyloid. PHF-tau was labeled with an antibody to phosphorylated tau, AT8 (1:2000, Thermoscientific). Total number of neurofibrillary tangle cell counts within a defined area was annotated by experienced raters using stereological mapping software (StereoInvestigator), which was converted to a density measure for tangles per square millimeter [19]. Region-specific β-amyloid load, and separately PHFtau tangle density, was square-root transformed and averaged across the 8 regions to obtain a composite. Pathologic AD diagnosis was determined based on the National Institute on Aging and Alzheimer’s Association (NIA-AA) research framework for AD [14].

Lewy bodies were identified with an antibody to α-synuclein (Zymed LB 509; 1:50; pSyn, 1:20,000; Wako Chemicals) [32]. Lewy bodies in amygdala, substantia nigra, entorhinal, anterior cingulate, midfrontal, middle temporal, or inferior parietal cortex were recorded and summarized as a binary variable (i.e., present vs absent). Classification of LATE-NC follows the recommendations by the LATE-NC working group with modification [20, 33]. TDP-43 cytoplasmic inclusions were identified with a phosphorylated monoclonal TAR5P-1D3 anti-TDP-43 antibody (pS409/410; 1:100) in amygdala, entorhinal cortex, hippocampus, midfrontal, middle temporal, anterior temporal tip, and inferior orbital frontal cortex. The inclusion ratings were summarized into 4 stages, i.e., no inclusion, localized to amygdala only, extension to the hippocampus or entorhinal cortex, and extension to the neocortex [1]. Hippocampal sclerosis refers to severe neuronal loss and gliosis of CA1 and subiculum, and was identified using 6 μm section of mid-hippocampus stained with hematoxylin and eosin (H&E) [31]. PART is characterized by neurofibrillary tangles confined to limbic regions (i.e., Braak stage ≤ 4) with minimal β-amyloid deposition (i.e., Thal stage ≤ 2) [10].

Chronic macroscopic infarcts were recorded during the gross examination and confirmed with histology. Chronic microinfarcts were identified using H&E stained sections from a minimum of nine regions (i.e., midfrontal, middle temporal, entorhinal, hippocampal, and inferior parietal cortex, anterior cingulate, anterior basal ganglia, thalamus and mid brain) [3]. Both infarcts were analyzed as binary variables.

A 4-level semi-quantitative rating (none, mild, moderate, and severe) was used to measure each of three vessel diseases. Meningeal and parenchymal vessels in midfrontal, midtemporal, parietal, and calcarine cortex were assessed for CAA [7]. Large vessel atherosclerosis was assessed by visually inspecting cerebral arteries and their proximal branches at the Circle of Willis [2]. Small vessel arteriolosclerosis was assessed using H&E stained sections of the basal ganglia [9].

### Plasma p-tau quantification

Blood samples were collected in 2-ml EDTA tubes by phlebotomists or nurses skilled in venipuncture during the home visits. Samples were transported to the Rush Alzheimer’s Disease Center laboratory. After centrifugation, samples were aliquoted, and stored in − 80 °C freezers. Plasma p-tau181 and p-tau217 were quantified on the MSD platform (Meso Scale Discovery) using immunoassays developed Lilly Research Laboratories, as previously described [16, 34]. Specifically, samples were analyzed in duplicates according to the published protocols with biotinylated-IBA493 (p-tau217) and biotinylated-IBA406 (p-tau181) used as capture antibodies and SULFO-TAG-4G10-E2 as the detector [17, 37]. The assays were calibrated with a synthetic p-tau217 and p-tau181 peptides. The average inter-assay coefficient of variation (CV) for 3 quality control samples included in every run were 7.4% and 5.1% in the p-tau217 and p-tau181 assays, respectively; the average intra-assay CVs were 6.2% and 4.8%, respectively. Three samples fell below the detection level for p-tau181 and 31 samples for p-tau217. These samples were included in the analyses, and exclusion of the samples with low detection level did not change the results.

### Statistical analysis 

Mean (SD), N (%), or median (interquartile range) summarized the key characteristics of the study participants. Bivariate correlations between plasma p-tau and continuous variables of interest (e.g., brain β-amyloid load) were assessed using Pearson’s *r*. Group difference in plasma p-tau was tested using analysis of variance (ANOVA). Multivariable linear or logistic regression models examined the associations of plasma p-tau with AD and other common neuropathologic indices. These models were adjusted for age, sex and education, and in secondary analyses we also included *APOE* ε4, and separately the time interval between blood collection and death. As the plasma p-tau measures were log2 transformed, the resulting regression coefficients estimated the difference in the outcome with every one-fold increase in the plasma p-tau level.

The receiver operating characteristic (ROC) curves were used to assess the accuracy for classifying AD relative to non-AD, and separately PART. The ROC curve traces the true positive rate (i.e., sensitivity) against the false positive rate (i.e., 1-specificity) with each observed plasma p-tau value as the threshold for classification. The areas under the curve (AUC) captures the overall model performance. A typical AUC falls between 0.5 (i.e., a random guess) and 1 (i.e., a perfect classification). Hence, the greater an AUC, the more accurate a model classifies a binary outcome. Statistical comparisons of AUCs between the models for ptau-181 and ptau-217 were conducted via bootstrapping. Briefly, a random sample, with a same N as the original data, was selected with replacement from the original dataset. Statistical models were run separately for plasma ptau-181 and ptau-217 using this bootstrap sample, and the difference between the resulting 2 AUCs was obtained. The process was repeated 500 times, and the result approximates a sampling distribution for the paired difference. A 95% bootstrapped confidence interval (CI) was computed by taking the 2.5th and 97.5th percentiles of the sampling distribution. If the 95% CI fails to cover zero, it would indicate a statistically significant difference between the AUCs.

## Results

### Neuropathologic characteristics of the study participants

**Fig. 1:**
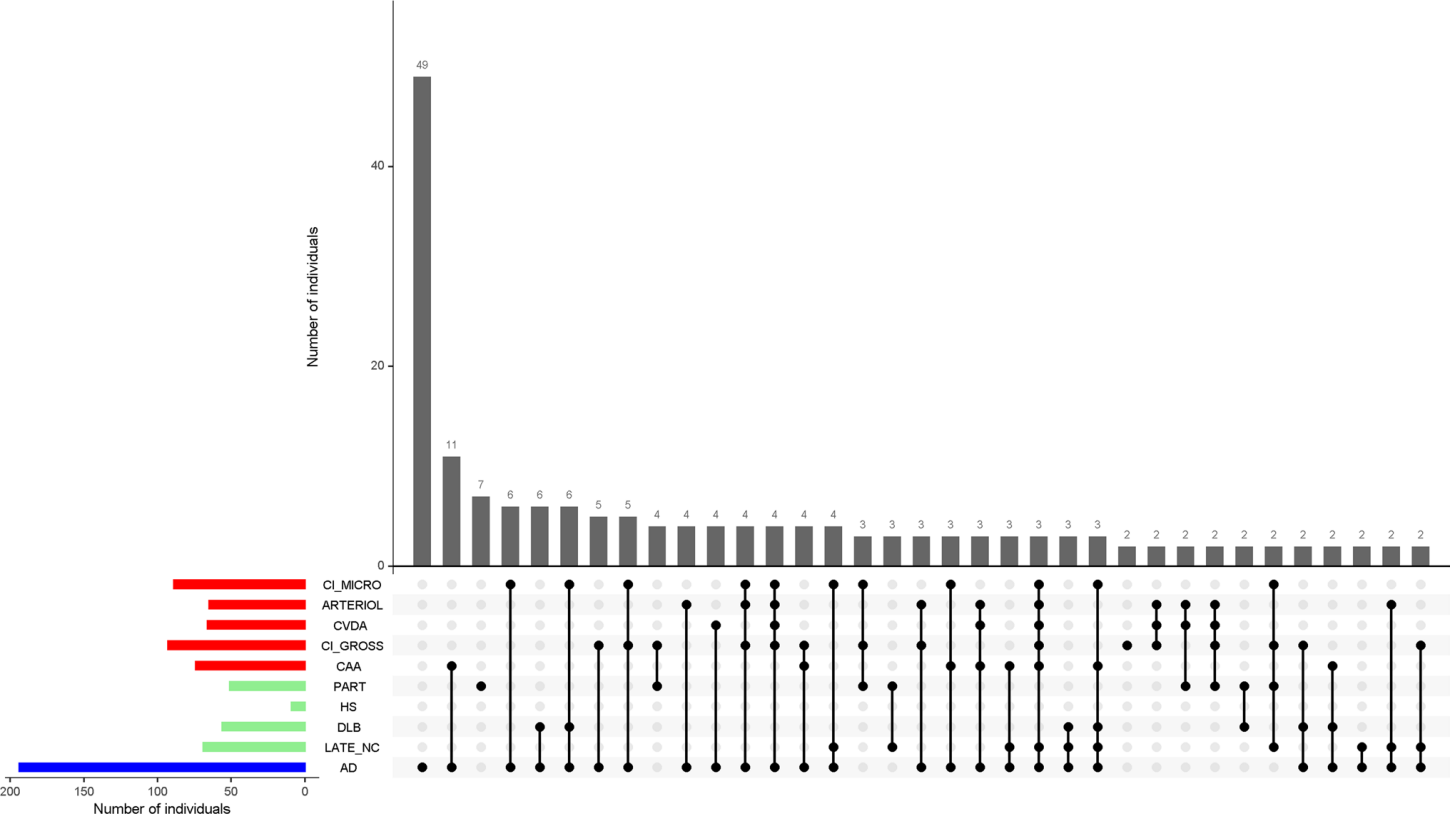
shows the burden of mixed pathologies in the aging brain. The bar chart on the lower left corner shows the frequencies of individual neuropathologic indices investigated in this study. The connected black dots on the x-axis indicate the specific combination of neuropathology represented (top 35 combinations shown). The histograms in the main panel show the frequencies of mixed neuropathologies, ordered by their frequency. The height of each bar corresponds to the number of persons with each combination

The study participants died at a mean age of 91 years (SD: 5.6 years). Due to the balanced design in case selection, individuals diagnosed with NCI, MCI, and Alzheimer’s dementia proximate to death were evenly distributed. On neuropathologic evaluation, over 70% of the individuals met the NIA-AA criteria for pathologic AD, approximately 40% had a non-AD degenerative condition including Lewy bodies, LATE-NC or hippocampal sclerosis, and 70% had cerebrovascular conditions of infarcts or vessel diseases (Table 1). We identified 52 individuals with possible or definite PART. Mixed pathologies were common (Fig. 1). In particular, of the individuals who were diagnosed with pathologic AD (*N* = 194), three quarters had co-existing degenerative and/or vascular conditions.

### Plasma p-tau and Alzheimer’s pathologies

Plasmas used for p-tau measurement in this study were collected on average 1.4 years before death (SD: 1.9 years). P-tau181 and p-tau217 were highly correlated, with a Pearson *r* of 0.92. We first examined the plasma p-tau measures in relation to the continuous measures for brain β-amyloid and PHFtau tangles. Plasma p-tau181 was moderately correlated with brain β-amyloid load (Pearson’s *r*: 0.38) and PHFtau tangles density (Pearson’s *r*: 0.41). The Pearson’s *r*s for plasma p-tau217 were 0.54 and 0.56 for β-amyloid and tangles, respectively (eFig. 1). Comparisons using 500 bootstrapped samples suggest that the correlations with AD pathologies were significantly higher for p-tau217 than p-tau181. Specifically, the 95% bootstrapped CI for the difference of Pearson’s *r*s was between 0.10 and 0.23 for the correlations with β-amyloid, and between 0.09 and 0.20 for the correlations with tangles.

In separate regression analyses that controlled for demographics, higher plasma p-tau181 and p-tau217 level were both associated with higher burdens of β-amyloid and PHFtau tangles (Table 2). Prior evidence suggests that increase in fluid levels of ptau-181 and ptau217 are induced by β-amyloid [36], therefore, we examined the extent to which the association of β-amyloid with PHFtau tangles is attributable to plasma ptau-181 and, separately, ptau-217. Path analyses partitioned the total effect of β-amyloid on PHFtau tangles into a direct effect and an indirect effect through plasma ptau, and standardized path coefficients quantified the proportion of the indirect effect (Table 3). In the model for ptau-181, the total standardized effect of β-amyloid on PHFtau tangles was 0.347 (Standard error [SE] = 0.062, *p* < 0.001). A majority of this total effect (65%) represented a direct effect (β = 0.225; SE = 0.067, *p* < 0.001), and the remaining 35% was the indirect effect through plasma p-tau181 (β = 0.122, SE = 0.032, *p* < 0.001). By contrast, almost all the total effect of β-amyloid on PHFtau tangles was attributable to the indirect effect through plasma p-tau217 (β = 0.281; SE = 0.045, *p* < 0.001), and the direct effect was not significant (β = 0.066; SE = 0.070, *p* = 0.340). The results were robust to *APOE* ε4 and the time interval between blood collection and death (eTables 1–4).

**Table 2:** Plasma p-tau with common neuropathologies

Neuropathologies	p-tau181			p-tau217		
	Estimate	SE	*p*	Estimate	SE	*p*
β-Amyloid^†^	0.469	0.083	< .001	0.491	0.055	< .001
Tangles^†^	0.534	0.082	< .001	0.538	0.055	< .001
Lewy bodies^‡^	0.183	0.182	0.313	0.226	0.133	0.089
LATE-NC^‡^	0.034	0.144	0.812	0.056	0.104	0.592
Gross infarcts^‡^	− 0.167	0.169	0.325	− 0.172	0.120	0.153
Microinfarcts^‡^	0.110	0.160	0.494	0.031	0.116	0.791
CAA^‡^	0.468	0.145	0.001	0.469	0.107	< .001
Atherosclerosis^‡^	− 0.108	0.142	0.448	-0.001	0.102	0.989
Arteriolosclerosis^‡^	0.040	0.139	0.775	0.092	0.100	0.357

For plasma p-tau181 and p-tau217 separately, each set of statistics (Estimate, SE, and *p*) came from a linear^†^ or logistic^‡^ regression models with each neuropathologic index as a cross-sectional outcome. All the models were controlled for age, sex and education. We did not examine the association with hippocampal sclerosis due to a small number (*N* = 10)

**Table 3:** Plasma p-tau in the association of brain β-amyloid with PHFtau tangles

Plasma ptau-181						Plasma ptau-217					
Path			Estimate	SE	*p*	Path			Estimate	SE	*p*
Tangles	←	Age	0.042	0.063	0.505	Tangles	←	Age	0.033	0.059	0.579
Tangles	←	Male sex	− 0.077	0.063	0.226	Tangles	←	Male sex	− 0.069	0.059	0.240
Tangles	←	Education	0.003	0.064	0.959	Tangles	←	Education	− 0.0003	0.059	0.997
Tangles	←	ptau-181	0.325	0.066	< .001	Tangles	←	ptau-217	0.524	0.063	< .001
Tangles	←	β-amyloid	0.225	0.067	< .001	Tangles	←	β-amyloid	0.066	0.069	0.340
β-amyloid	←	Age	0.004	0.071	0.950	β-amyloid	←	Age	0.004	0.071	0.950
β-amyloid	←	Male sex	0.065	0.071	0.359	β-amyloid	←	Male sex	0.065	0.071	0.359
β-amyloid	←	Education	0.049	0.071	0.489	β-amyloid	←	Education	0.049	0.071	0.489
ptau-181	←	Age	0.076	0.065	0.243	ptau-217	←	Age	0.065	0.060	0.274
ptau-181	←	Male sex	− 0.049	0.066	0.459	ptau-217	←	Male sex	− 0.044	0.060	0.461
ptau-181	←	Education	0.102	0.065	0.117	ptau-217	←	Education	0.070	0.060	0.241
ptau-181	←	β-amyloid	0.375	0.061	< .001	ptau-217	←	β-amyloid	0.536	0.051	< .001

In a secondary analysis, we explored whether the indirect effect of β-amyloid on PHFtau tangles through plasma p-tau may differ by dementia status. We repeated the path analysis separately for individuals without dementia and those with dementia (eTable 5). Among the non-demented, in the model for ptau-181, we observed a larger proportion of the direct effect of β-amyloid on PHFtau tangles (85%), and the indirect effect through ptau-181 was nominal (15%). By contrast, in the model for ptau-217, approximately half of the effect of β-amyloid on PHFtau tangles was direct and half was through ptau-217. Among the demented, in the model for ptau-181, 66% of the total effect of β-amyloid on PHFtau tangles was through ptau-181, and in the model for ptau-217, all of the effect of β-amyloid on PHFtau tangles was through ptau-217. Together, these results indicate that regardless of dementia status, compared to plasma p-tau181, ptau-217 was more involved in the relationship between β-amyloid and tangles. Separately, the magnitude of the indirect effect of plasma p-tau seemed weaker among older adults without dementia. Due to small sample sizes in the stratified analyses, the latter results need to be interpreted with caution and further validation is warranted.

Consistent with the results for quantitative AD pathologic indices, higher levels of plasma p-tau were also associated with greater risk of a pathologic diagnosis of AD. Specifically, the odds of having an AD diagnosis were tripled with every one-fold increase in plasma p-tau181 (odd ratio [OR] 3.09, 95% CI 1.95–4.91), and were almost quadrupled with every one-fold increase in plasma p-tau217 (OR 3.76, 95% CI 2.52–5.63). Further, the AUC was 0.76 for the model with p-tau181, and 0.83 for the model with p-tau217. The paired difference in the AUCs using 500 bootstrapped samples had a 95% CI between 0.04 and 0.11, suggesting that plasma p-tau217 has higher accuracy in differentiating AD compared to plasma p-tau181. In a secondary analysis that controlled for demographics and *APOE* ε4, p-tau181 (OR = 2.48, 95% CI = 1.54–4.00) and p-tau217 (OR = 3.76, 95% CI = 2.21–5.08) remained strongly associated with AD. The results were also unchanged if we controlled for the time interval between blood collection and death, where the OR was 3.10 for p-tau181 (95% CI = 1.95–4.93) and 3.79 for p-tau217 (95% CI = 2.53–5.67).

### Plasma p-tau and non-AD neuropathologies

We did not observe a significant association of plasma p-tau with other non-AD neuropathologies, with the exception of CAA (Table 2, eTables 1 and 2). With every one-fold increase in plasma p-tau181, and separately p-tau217, the odds of having more severe CAA were about 60% higher without adjusting for *APOE* ε4, and about 50% higher after the adjustment. As β-amyloid was also positively correlated with CAA, we repeated the analysis by including β-amyloid in the model. The strength of associations was attenuated, but higher plasma p-tau217 remained associated with higher burden of CAA (eTable 6).

### Plasma p-tau for differentiating AD from PART

Given that little is known about the extent to which p-tau may be specific to AD versus PART, we examined whether plasma p-tau was able to differentiate AD from PART. Since the overlap between PART and AD predominantly occurs in Braak stage 3 or 4, we restricted our analysis to this subsample (*N* = 160) where approximately 80% had AD and 20% had PART (eTable 7). The two groups were similar in demographics. The AD group had more *APOE* ε4 carriers, and the PART group had more individuals with macroscopic infarcts. Of note, we did not observe significant difference in PHFtau tangles density between AD and PART. Compared to PART, the average plasma p-tau levels were higher in AD. The mean log2 transformed p-tau181 level was 1.86 in AD and 1.34 in PART (*t*_156_ = − 3.71, *p* < 0.001), suggesting that the plasma p-tau181 level was about 40% higher in AD than PART. Similarly, the mean log2 transformed p-tau217 was − 1.23 in AD and − 2.18 in PART (*t*_63.0_ = − 6.54, *p* < 0.001), suggesting that the p-tau217 level was nearly twice higher in AD than PART. Among individuals with PART, we did not observe a p-tau level difference across the Braak stages (eFigure 2).

**Fig. 2:**
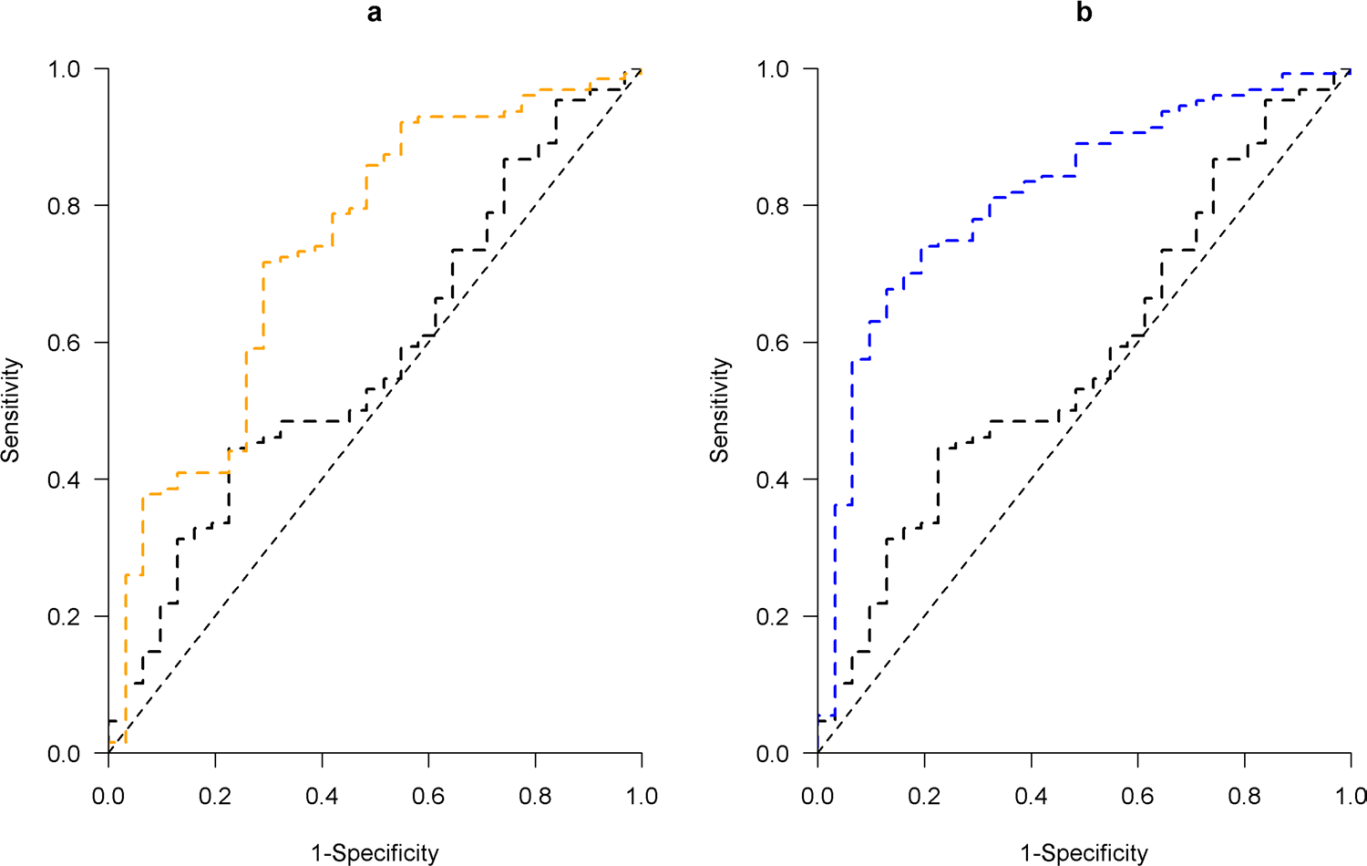
shows the areas under the receiver operating characteristic (ROC) curves for classifying AD versus PART. Within each panel, the black curve represents the estimated ROC for the model with demographics only. The orange curve on **a** represents the ROC for p-tau181, and the blue curve on **b** represents the ROC for p-tau217

In logistic regression analyses adjusted for demographics, both plasma p-tau measures were associated with greater odds of AD. With every one-fold increase in plasma p-tau181, the OR of having an AD diagnosis relative to PART was 3.49 (95% CI 1.69–7.18), and the corresponding OR for p-tau217 was 4.67 (95% CI 2.38–9.15). Further, compared to p-tau181, p-tau217 showed a higher AUC (0.82 versus 0.74) in classifying AD from PART (Fig. 2), and the 95% bootstrapped CI for the paired difference in AUC was between 0.03 and 0.13 and did not cover zero. The results were similar after adjustment for *APOE* ε4, where the OR of having AD over PART was 2.67 (95% CI 1.23–5.76) for p-tau181 and 3.94 (95% CI 1.92–8.07) for p-tau217. Nor did adjustment for the time interval between blood collection and death change the results, and the ORs were 3.43 (95% CI = 1.66–7.08) for p-tau181 and 4.65 (95% CI = 2.37–9.13) for p-tau217.

Next, we conducted a stratified analysis to examine whether the results for plasma p-tau and PART differ by dementia status. In this subsample of 160 individuals, about 3 quarters died without developing dementia. Among the non-demented group, both higher p-tau181 (OR 2.95, 95% CI 1.22–7.13) and p-tau217 (OR 3.66, 95% CI 1.72–7.79) were associated with higher odds of having AD over PART. The corresponding AUC was 0.80 for p-tau181 and 0.83 for p-tau217. We observed similar associations for the dementia group. However, the small sample size (*N* = 41) introduced very wide confidence intervals, therefore we chose not to report the results as these statistics may not be stable. Overall, our data provide preliminary evidence that plasma p-tau association with PART does not differ by dementia status.

### Examining whether plasma p-tau levels vary in mixed AD pathologies

**Fig. 3:**
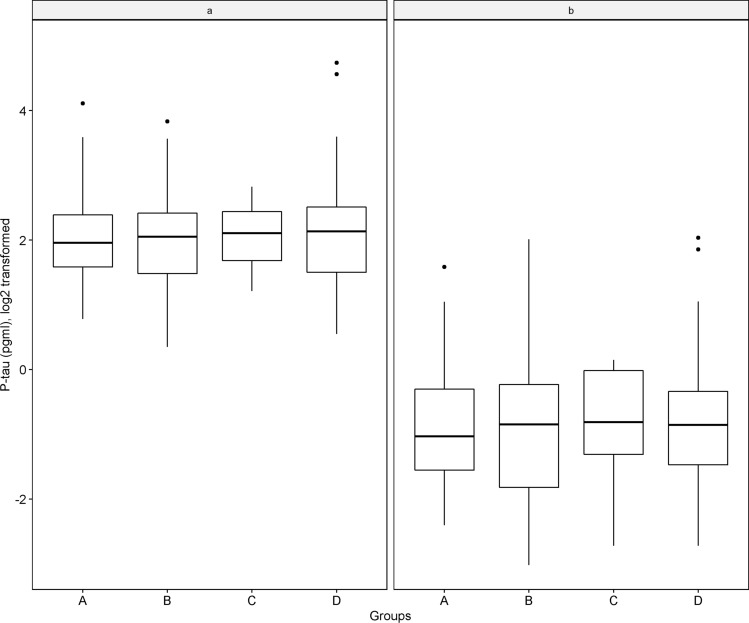
shows the levels of plasma p-tau measures by mixed AD groups (A: AD only, B: AD mixed with vascular conditions, C: AD mixed with other degenerative conditions, and D: AD mixed with vascular and other degenerative conditions). Each panel, with plasma p-tau181 on the left (**a**) and p-tau217 on the right (**b**), is a boxplot with the height of each box representing the interquartile range and line segment inside the box representing the median

As a majority of individuals with AD had mixed pathologies, we investigated the extent to which plasma p-tau levels vary between mixed AD pathologies. Specifically, we compared p-tau levels among individuals with AD only (*N* = 49), AD mixed with other degenerative conditions (*N* = 12), AD mixed with vascular conditions (*N* = 70), and AD mixed with both other degenerative and vascular conditions (*N* = 63). The results from the ANOVA suggested that neither plasma p-tau181 (*F*_3,189_ = 0.134, *p* = 0.940) nor p-tau217 (*F*_3,189_ = 0.153, *p* = 0.927) differed between AD only and various mixed AD groups (Fig. 3).

## Discussion

In this work, by leveraging clinicopathologic data, blood and brain biospecimens from over 250 community-dwelling older adults, we examined the associations of plasma p-tau181 and p-tau217 with AD and other common neuropathologies in aging brain. Both plasma measures were associated with greater odds of AD, with p-tau217 having higher accuracy. The plasma p-tau measures were almost exclusively associated with AD pathologic indices except for CAA. Further, among individuals with comparable Braak stages and PHFtau burden, plasma p-tau217 differentiated AD from PART. For both biomarkers, we did not observe a level difference between individuals with AD alone versus those with mixed AD pathologies. These findings advance our understanding of the connections between plasma p-tau and key neuropathologic drivers of ADRD.

Here, we confirmed that both plasma p-tau181 and p-tau217 are strongly associated with AD neuropathologic indices of β-amyloid and PHFtau tangles. Interestingly, while the correlation between the two p-tau measures was over 0.9, the accuracy of differentiating autopsy-confirmed AD was higher for p-tau217 than p-tau181 (AUC of 0.83 vs 0.76). These AUC statistics are largely consistent with prior literature. For example, one prior study reported that the AUC of differentiating AD (i.e., intermediate to high likelihood based on the NIA-Reagan criteria) was 0.89 for plasma p-tau217 and 0.72 for p-tau181 [34]. A similar result was reported when comparing p-tau measures from CSF [18, 23]. The underlying biology for such difference, if present, is unclear. It is possible that, compared with p-tau181, p-tau217 preferentially reflects tau aggregation that contain PHF in AD. This hypothesis is supported by a comparative study which showed that the antibody AT100 which detects p-tau217 captured more neurofibrillary tangles than the antibody AT270 which detects p-tau181. In our data, we observed a higher correlation between PHFtau tangles and plasma p-tau217 than p-tau181.

We showed that plasma p-tau has potential for differentiating AD from PART, and in particular the AUC for the model with p-tau217 was above 0.8. Importantly, this result was based on a subsample of individuals with same Braak stages (i.e., 3 or 4) where AD and PART largely overlap. To our knowledge, this is the first time that the field has ever investigated the relationship between plasma p-tau and AD versus PART. The observation that plasma p-tau differentiates AD from PART in older adults with comparable tangles burden suggests that p-tau181 and p-tau217 are more related to amyloid accumulation. As discussed below, this is also supported by plasma p-tau associations with CAA. Separately, in the tau biomarker community, both biofluid and PET, there are unresolved questions on how to best identify PART. For example, ^18^F-flortaucipir tau PET tends to only recognize advanced tau stages (i.e., Braak 5 or 6), but ^18^F-MK-6240 tau PET, which has higher sensitivity to tau, may also recognize Braak stage 3 or 4. Thus, combining both ^18^F-MK-6240 tau PET and plasma ptau217 may increase the specificity for detecting PART. Notably, our preliminary data suggest that there is no evidence of an increase in plasma p-tau level across severity of PART.

Regarding non-AD pathologies, we observed that neither plasma p-tau181 nor p-tau217 was associated with non-AD degenerative or vascular conditions, with the exception of CAA. Interestingly, the plasma p-tau association with CAA was not fully explained by brain β-amyloid burden, a result that may be consistent with a recent report that amyloid angiopathy interacts with neuritic plaques to accelerate tau burden [38]. We also note that plasmas p-tau levels did not differ between individuals with AD only and those with mixed AD pathologies. The implications are twofold. First, these findings complement prior studies on the diagnostic utility of plasma p-tau for discriminating AD from other neurodegenerative conditions, and lend additional support that these plasma biomarkers are specific to AD. The specificity of AD is of utmost importance, because it is a prerequisite for any diagnostic marker that will be used to identify AD before initiating disease-modifying therapies, like β-amyloid immunotherapies, in clinical practice and trials. A unique feature of this current work is that, rather than relying on clinical criteria for identifying individuals with various degenerative disorders, we examined specific neuropathologic indices that characterize brain conditions known to cause cognitive impairment and dementia.

Second, these findings highlight that β-amyloid is heavily implicated in plasma p-tau. We observed significant correlations between plasma p-tau181, separately p-tau217, and brain β-amyloid burden. Further, while the plasma biomarkers and β-amyloid were both associated with tangle pathology, the association of brain β-amyloid load with PHFtau tangle density was attenuated and no longer significant after plasma p-tau217 was included in the model. This is congruent with our previous results showing that increase in soluble levels of p-tau fully mediates the associations between amyloid plaques and PHFtau [24]. Further, recent work has shown that amyloid-induced increases in soluble p-tau are strongly associated with accumulation of insoluble tau aggregates and cognitive decline over time, especially in the early stages of AD [36].

A unique strength of our study is that all participants are community-based and agreed to brain donation. The autopsy rate is over 85%, and multiple ADRD neuropathologies including AD, non-AD neurodegenerative, and cerebrovascular pathologies were quantified in a relatively large number of older persons. Systematic uniform postmortem assessments are blinded to the clinical data. This allows us to interrogate the associations of plasma p-tau with single as well as mixed neuropathologies. An important limitation is that all the participants in the study were older Whites. Plasma p-tau data collection for diverse populations is just emerging [8]. The current study focused on p-tau181 and p-tau217, and other tau measures, including p-tau231 [28], brain-derived tau [11] or glial fibrillary acidic protein (GFAP) [35], may also be promising biomarkers for AD. Separately, the current plasma p-tau measures were restricted to one timepoint proximate to death. More extensive studies are warranted to examine the longitudinal change in the plasma p-tau level as people age and its relation to neuropathologies. All ROSMAP participants are free of known dementia at baseline and a majority of them have no cognitive impairment. They underwent annual clinical evaluations with longitudinal blood collection, and all agreed to brain autopsy. This design makes ROSMAP well suited for addressing these important questions, and such investigations are planned.

## Supplementary Information

Below is the link to the electronic supplementary material.

Supplementary file1 (DOCX 326 KB)

## Data Availability

Data used for this study can be requested for research purposes via the RADC Research Resource Sharing Hub at https://www.radc.rush.edu/.
