# Supplementary material for: Plasma p-tau181 and p-tau217 in discriminating PART, AD and other key neuropathologies in older adults
Source: Acta Neuropathol. 2023 Apr 9;146(1):1–11. doi: 10.1007/s00401-023-02570-4 (PMC10261204; doi:10.1007/s00401-023-02570-4)

**Plasma p-tau181 and p-tau217 in discriminating PART, AD and other key neuropathologies in older adults**

**eTable 1 Plasma p-tau with common neuropathologies after adjustment for *APOE* ε4**

| Neuropathologies | p-tau181 | | | p-tau217 | | |
| --- | --- | --- | --- | --- | --- | --- |
|  | Estimate | SE | *p* | Estimate | SE | *p* |
| β-Amyloid^†^ | 0.397 | 0.084 | <.001 | 0.447 | 0.057 | <.001 |
| Tangles^†^ | 0.545 | 0.088 | <.001 | 0.574 | 0.059 | <.001 |
| Lewy bodies^‡^ | 0.136 | 0.191 | 0.475 | 0.193 | 0.141 | 0.172 |
| LATE-NC^‡^ | -0.048 | 0.154 | 0.759 | -0.004 | 0.112 | 0.974 |
| Gross infarcts^‡^ | -0.106 | 0.179 | 0.554 | -0.131 | 0.129 | 0.308 |
| Microinfarcts^‡^ | 0.084 | 0.172 | 0.627 | 0.002 | 0.127 | 0.990 |
| CAA^‡^ | 0.406 | 0.154 | 0.008 | 0.422 | 0.115 | <.001 |
| Atherosclerosis^‡^ | -0.037 | 0.150 | 0.804 | 0.065 | 0.110 | 0.555 |
| Arteriolosclerosis^‡^ | 0.040 | 0.147 | 0.788 | 0.083 | 0.107 | 0.438 |

For plasma p-tau181 and p-tau217 separately, each set of statistics (Estimate, SE, and *p*) came from a linear^†^ or logistic^‡^ regression models with each neuropathologic index as a cross-sectional outcome. All the models were controlled for age, sex, education, and *APOE* ε4. We did not examine the association with hippocampal sclerosis due to a small number (N=10).

**eTable 2 Plasma p-tau with common neuropathologies after adjustment for the time interval between blood collection and death**

| Neuropathologies | p-tau181 | | | p-tau217 | | |
| --- | --- | --- | --- | --- | --- | --- |
|  | Estimate | SE | *p* | Estimate | SE | *p* |
| β-Amyloid^†^ | 0.470 | 0.083 | <.001 | 0.490 | 0.055 | <.001 |
| Tangles^†^ | 0.527 | 0.082 | <.001 | 0.536 | 0.054 | <.001 |
| Lewy bodies^‡^ | 0.183 | 0.182 | 0.316 | 0.227 | 0.134 | 0.090 |
| LATE-NC^‡^ | 0.037 | 0.144 | 0.798 | 0.059 | 0.104 | 0.573 |
| Gross infarcts^‡^ | -0.157 | 0.170 | 0.357 | -0.164 | 0.121 | 0.173 |
| Microinfarcts^‡^ | 0.115 | 0.161 | 0.474 | 0.035 | 0.117 | 0.764 |
| CAA^‡^ | 0.464 | 0.145 | 0.001 | 0.467 | 0.107 | <.001 |
| Atherosclerosis^‡^ | -0.102 | 0.142 | 0.474 | 0.006 | 0.103 | 0.955 |
| Arteriolosclerosis^‡^ | 0.081 | 0.140 | 0.566 | 0.132 | 0.101 | 0.192 |

For plasma p-tau181 and p-tau217 separately, each set of statistics (Estimate, SE, and *p*) came from a linear^†^ or logistic^‡^ regression models with each neuropathologic index as a cross-sectional outcome. All the models were controlled for age, sex, education, and the time interval between blood collection and death. We did not examine the association with hippocampal sclerosis due to a small number (N=10).

**eTable 3 Plasma p-tau in the association of brain β-amyloid with PHFtau tangles after adjustment for *APOE* ε4**

| Plasma ptau-181 | | | | | | Plasma ptau-217 | | | | | |
| --- | --- | --- | --- | --- | --- | --- | --- | --- | --- | --- | --- |
| Path | | | Estimate | SE | *p* | Path | | | Estimate | SE | *p* |
| tangles | 🡨 | age | 0.009 | 0.065 | 0.895 | tangles | 🡨 | age | -0.002 | 0.060 | 0.968 |
| tangles | 🡨 | male sex | -0.079 | 0.064 | 0.216 | tangles | 🡨 | male sex | -0.075 | 0.059 | 0.210 |
| tangles | 🡨 | education | 0.008 | 0.065 | 0.904 | tangles | 🡨 | education | 0.005 | 0.061 | 0.929 |
| tangles | 🡨 | *APOE* ε4 | -0.087 | 0.070 | 0.213 | tangles | 🡨 | *APOE* ε4 | -0.104 | 0.065 | 0.110 |
| tangles | 🡨 | ptau-181 | 0.329 | 0.067 | <.001 | tangles | 🡨 | ptau-217 | 0.531 | 0.064 | <.001 |
| tangles | 🡨 | β-amyloid | 0.268 | 0.070 | <.001 | tangles | 🡨 | β-amyloid | 0.111 | 0.072 | 0.124 |
| β-amyloid | 🡨 | age | 0.067 | 0.069 | 0.331 | β-amyloid | 🡨 | age | 0.067 | 0.069 | 0.331 |
| β-amyloid | 🡨 | male sex | 0.073 | 0.068 | 0.283 | β-amyloid | 🡨 | male sex | 0.073 | 0.068 | 0.283 |
| β-amyloid | 🡨 | education | -0.010 | 0.070 | 0.882 | β-amyloid | 🡨 | education | -0.010 | 0.070 | 0.883 |
| β-amyloid | 🡨 | *APOE* ε4 | 0.341 | 0.067 | <.001 | β-amyloid | 🡨 | *APOE* ε4 | 0.342 | 0.067 | <.001 |
| ptau-181 | 🡨 | age | 0.089 | 0.067 | 0.184 | ptau-217 | 🡨 | age | 0.076 | 0.061 | 0.215 |
| ptau-181 | 🡨 | male sex | -0.048 | 0.066 | 0.466 | ptau-217 | 🡨 | male sex | -0.039 | 0.061 | 0.524 |
| ptau-181 | 🡨 | education | 0.099 | 0.067 | 0.142 | ptau-217 | 🡨 | education | 0.066 | 0.061 | 0.285 |
| ptau-181 | 🡨 | *APOE* ε4 | 0.107 | 0.072 | 0.136 | ptau-181 | 🡨 | *APOE* ε4 | 0.098 | 0.066 | 0.135 |
| ptau-181 | 🡨 | β-amyloid | 0.375 | 0.061 | <.001 | ptau-217 | 🡨 | β-amyloid | 0.505 | 0.056 | <.001 |

**eTable 4 Plasma p-tau in the association of brain β-amyloid with PHFtau tangles after adjustment for the time interval between blood collection and death**

| Plasma ptau-181 | | | | | | Plasma ptau-217 | | | | | |
| --- | --- | --- | --- | --- | --- | --- | --- | --- | --- | --- | --- |
| Path | | | Estimate | SE | *p* | Path | | | Estimate | SE | *p* |
| tangles | 🡨 | age | 0.044 | 0.063 | 0.486 | tangles | 🡨 | age | 0.034 | 0.059 | 0.558 |
| tangles | 🡨 | male sex | -0.076 | 0.063 | 0.230 | tangles | 🡨 | male sex | -0.069 | 0.059 | 0.245 |
| tangles | 🡨 | education | 0.005 | 0.063 | 0.939 | tangles | 🡨 | education | 0.001 | 0.059 | 0.985 |
| tangles | 🡨 | time | 0.070 | 0.063 | 0.265 | tangles | 🡨 | time | 0.070 | 0.058 | 0.232 |
| tangles | 🡨 | ptau-181 | 0.322 | 0.066 | <.001 | tangles | 🡨 | ptau-217 | 0.522 | 0.063 | <.001 |
| tangles | 🡨 | β-amyloid | 0.231 | 0.067 | <.001 | tangles | 🡨 | β-amyloid | 0.072 | 0.069 | 0.298 |
| β-amyloid | 🡨 | age | 0.003 | 0.071 | 0.968 | β-amyloid | 🡨 | age | 0.003 | 0.071 | 0.968 |
| β-amyloid | 🡨 | male sex | 0.064 | 0.071 | 0.368 | β-amyloid | 🡨 | male sex | 0.064 | 0.071 | 0.368 |
| β-amyloid | 🡨 | education | 0.048 | 0.071 | 0.501 | β-amyloid | 🡨 | education | 0.048 | 0.071 | 0.501 |
| β-amyloid | 🡨 | time | -0.070 | 0.071 | 0.322 | β-amyloid | 🡨 | time | -0.070 | 0.071 | 0.322 |
| ptau-181 | 🡨 | age | 0.077 | 0.065 | 0.238 | ptau-217 | 🡨 | age | 0.066 | 0.060 | 0.270 |
| ptau-181 | 🡨 | male sex | -0.048 | 0.066 | 0.463 | ptau-217 | 🡨 | male sex | -0.044 | 0.060 | 0.464 |
| ptau-181 | 🡨 | education | 0.103 | 0.065 | 0.115 | ptau-217 | 🡨 | education | 0.071 | 0.060 | 0.239 |
| ptau-181 | 🡨 | time | 0.032 | 0.065 | 0.622 | ptau-181 | 🡨 | time | 0.020 | 0.060 | 0.736 |
| ptau-181 | 🡨 | β-amyloid | 0.378 | 0.061 | <.001 | ptau-217 | 🡨 | β-amyloid | 0.537 | 0.051 | <.001 |

time: the time interval between blood collection and death

**eTable 5aPlasma p-tau in the association of brain β-amyloid with PHFtau tangles (Full sample)**

| Plasma ptau-181 | | | | | | Plasma ptau-217 | | | | | |
| --- | --- | --- | --- | --- | --- | --- | --- | --- | --- | --- | --- |
| Path | | | Estimate | SE | *p* | Path | | | Estimate | SE | *p* |
| tangles | 🡨 | ptau-181 | 0.333 | 0.065 | <.001 | tangles | 🡨 | ptau-217 | 0.531 | 0.062 | <.001 |
| tangles | 🡨 | β-amyloid | 0.217 | 0.067 | 0.001 | tangles | 🡨 | β-amyloid | 0.057 | 0.069 | 0.409 |
| ptau-181 | 🡨 | β-amyloid | 0.378 | 0.061 | <.001 | ptau-217 | 🡨 | β-amyloid | 0.537 | 0.051 | <.001 |

For model parsimony in these analyses, we did not adjust for demographics. In the full sample, the proportions of indirect effect due to plasma p-tau181, and separately p-tau217, are similar to the models with adjustment for demographics (Table 3).

**eTable 5b Plasma p-tau in the association of brain β-amyloid with PHFtau tangles (Non dementia)**

| Plasma ptau-181 | | | | | | Plasma ptau-217 | | | | | |
| --- | --- | --- | --- | --- | --- | --- | --- | --- | --- | --- | --- |
| Path | | | Estimate | SE | *p* | Path | | | Estimate | SE | *p* |
| tangles | 🡨 | ptau-181 | 0.190 | 0.079 | 0.017 | tangles | 🡨 | ptau-217 | 0.349 | 0.082 | <.001 |
| tangles | 🡨 | β-amyloid | 0.322 | 0.077 | <.001 | tangles | 🡨 | β-amyloid | 0.202 | 0.085 | 0.017 |
| ptau-181 | 🡨 | β-amyloid | 0.304 | 0.077 | <.001 | ptau-217 | 🡨 | β-amyloid | 0.509 | 0.063 | <.001 |

**eTable 5c Plasma p-tau in the association of brain β-amyloid with PHFtau tangles (Dementia)**

| Plasma ptau-181 | | | | | | Plasma ptau-217 | | | | | |
| --- | --- | --- | --- | --- | --- | --- | --- | --- | --- | --- | --- |
| Path | | | Estimate | SE | *p* | Path | | | Estimate | SE | *p* |
| tangles | 🡨 | ptau-181 | 0.496 | 0.122 | <.001 | tangles | 🡨 | ptau-217 | 0.710 | 0.113 | <.001 |
| tangles | 🡨 | β-amyloid | 0.151 | 0.132 | 0.252 | tangles | 🡨 | β-amyloid | -0.038 | 0.132 | 0.776 |
| ptau-181 | 🡨 | β-amyloid | 0.599 | 0.085 | <.001 | ptau-217 | 🡨 | β-amyloid | 0.683 | 0.071 | <.001 |

**eTable 6 Plasma p-tau, brain β-amyloid and CAA**

|  | Model 1 | Model 2 | Model 3 | Model 4 | Model 5 |
| --- | --- | --- | --- | --- | --- |
| Age | 1.049 (0.998, 1.103) | 1.043 (0.992, 1.097) | 1.045 (0.993, 1.099) | 1.043 (0.992, 1.097) | 1.044 (0.993, 1.098) |
| Male Sex | 1.561 (0.867, 2.811) | 1.663 (0.923, 2.998) | 1.599 (0.886, 2.886) | 1.662 (0.920, 3.003) | 1.624 (0.898, 2.936) |
| Education | 1.052 (0.960, 1.152) | 1.038 (0.947, 1.138) | 1.041 (0.950, 1.141) | 1.037 (0.946, 1.136) | 1.039 (0.948, 1.139) |
| Amyloid | 1.436 (1.105, 1.866) | - | 1.320 (0.998, 1.746) | - | 1.172 (0.864, 1.589) |
| p-tau181 | - | 1.535 (1.115, 2.114) | 1.384 (0.982, 1.951) | - | - |
| p-tau217 | - | - | - | 1.570 (1.234,1.999) | 1.465 (1.107, 1.938) |

Each model is a multivariable logistic regression analysis with CAA as a 4-level ordinal outcome. Statistics in each cell are the odds ratio and the corresponding 95% confidence interval (in parenthesis).

**eTable 7 Characteristics of individuals with AD versus PART (N=160)**

|  | AD (N=129) | PART (N=31) | *p* |
| --- | --- | --- | --- |
| Age at death, years^†^ | 90.7 (5.6) | 90.7 (4.2) | 0.950 |
| Female^Δ^ | 86 (66.7%) | 20 (64.5%) | 0.820 |
| Education, years^†^ | 16.1 (3.6) | 15.1 (3.2) | 0.149 |
| *APOE* ε4 carriers^Δ^ | 31 (26.5%) | 1 (3.5%) | 0.005 |
| Dementia^Δ^ | 29 (22.5%) | 12 (38.7%) | 0.063 |
| β-amyloid load^§^ | 4.26 (2.28-6.50) | 0 (0-0.15) | <.001 |
| PHFtau tangle density^§^ | 4.36 (2.28-7.62) | 5.12 (2.96-7.04) | 0.404 |
| Lewy bodies^Δ^ | 22 (17.1%) | 8 (25.8%) | 0.262 |
| LATE-NC (Stage >1)^Δ^ | 29 (22.8%) | 7 (22.6%) | 0.990 |
| Chronic macroscopic infarcts^Δ^ | 36 (28.1%) | 19 (61.3%) | <.001 |
| Chronic microinfarcts^Δ^ | 36 (28.1%) | 14 (45.2%) | 0.067 |
| CAA (Moderate or Severe)^Δ^ | 36 (27.9%) | 5 (16.1%) | 0.177 |
| Atherosclerosis (Moderate or Severe)^Δ^ | 26 (20.2%) | 10 (32.2%) | 0.147 |
| Arteriolosclerosis (Moderate or Severe)^Δ^ | 26 (20.1%) | 6 (19.4%) | 0.920 |

^†^ Mean (Standard deviation); ^Δ^ N(%); ^§^ Median (Interquartile range); MMSE: Mini-mental state examination; LATE-NC: Limbic predominant age-related TDP-43 encephalopathy neuropathologic changes; CAA: Cerebral amyloid angiopathy.

Group difference was tested using t-test, chi-squared test, Fisher’s exact test, or non-parametric Wilcoxon rank sum test.

**eFigure 1 Plasma p-tau and Alzheimer’s neuropathologies**


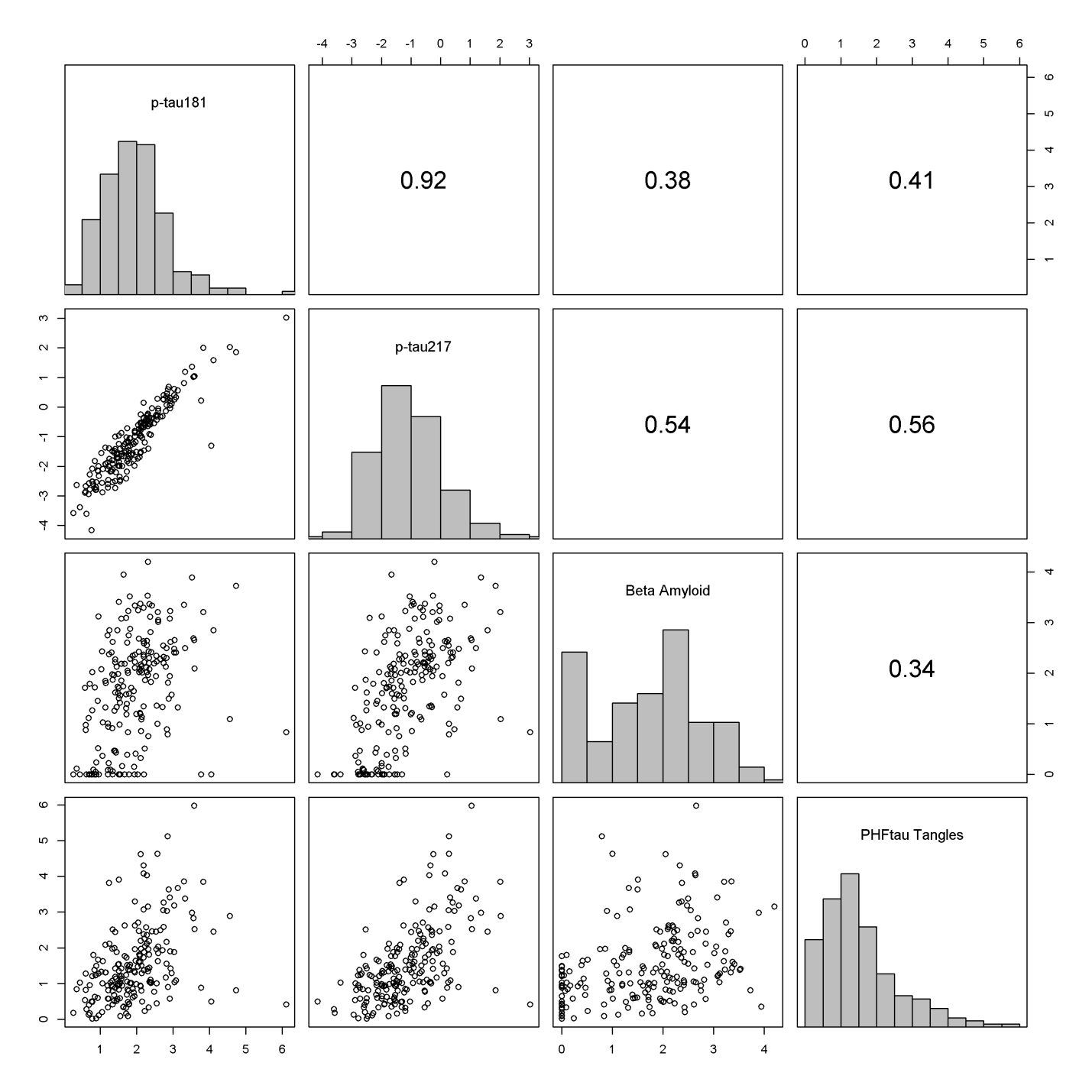


**eFigure 2 Plasma p-tau by Braak stages in PART**


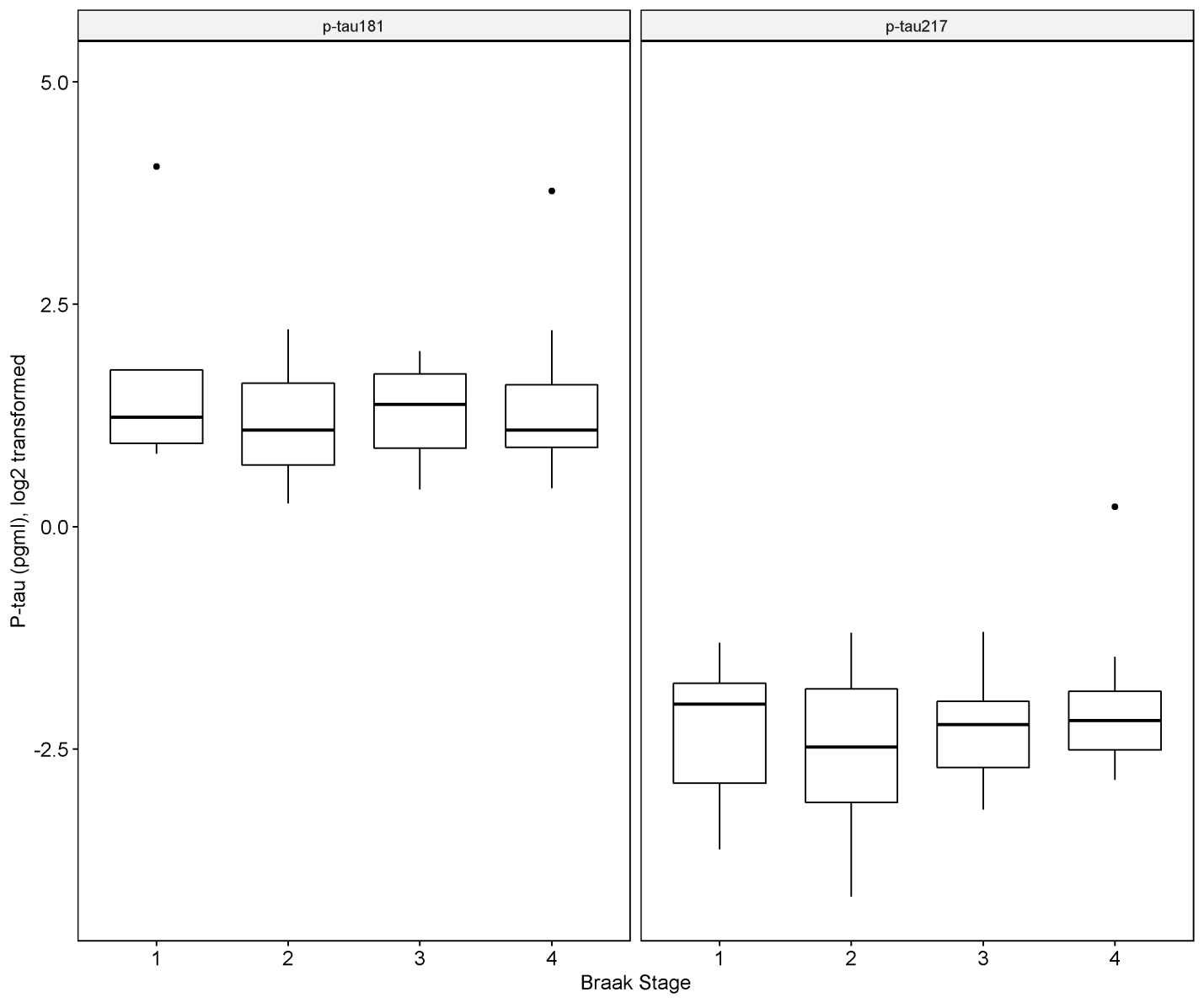

Supplement: Supplementary file 1 — Supplementary file1 (DOCX 326 KB) [file 401_2023_2570_MOESM1_ESM.docx]
